# A Case Report of a Successful Bilateral Lichtenstein Repair Using Elastic Tacks

**DOI:** 10.7759/cureus.85475

**Published:** 2025-06-06

**Authors:** Mekhaeel Shehata Fakhry Mekhaeel, Andrey Vitalevitch Protasov, Sameh Mohamed Salem

**Affiliations:** 1 Operative Surgery and Clinical Anatomy, Peoples’ Friendship University of Russia, Moscow, RUS

**Keywords:** bilateral inguinal hernia, inguinal hernia repair, lichtenstein technique, mesh fixation, mesh repair

## Abstract

This manuscript is dedicated to the examination of a case involving a 55-year-old male patient who has experienced bilateral inguinal hernias for a duration of three years. The patient was subsequently referred to our department from the outpatient clinic for the purpose of determining an appropriate surgical intervention. The patient presented with comorbidities including primary arterial hypertension, duodenal ulcer, chronic prostatitis, gastroesophageal reflux disease (GERD), chronic esophagitis, and occurrences of supraventricular tachycardia. Our research team elected to implement the Lichtenstein tension-free repair modification for both sides within a single surgical operation. Bilateral inguinal hernioplasty was performed successfully on both sides at the same surgical procedure, employing the Lichtenstein technique, which contributed to reducing the operative duration and postoperative complications regarding mesh implants secured with elastic absorbable tacks. The patient experienced no postoperative complications, such as neuralgia, seroma, foreign body sensation, or recurrence. The next five years were scheduled for follow-up every six months. The adoption of this technique yielded outcomes that met expectations.

## Introduction

For males, the lifetime risk of inguinal hernia is 27%, while for females, it is 3%. It is diagnosed by the protrusion of abdominal contents through the anterolateral abdominal wall's muscle and fascial layers [1]. Inguinal hernia formation is associated with environmental, genetic, and behavioral factors [2], as well as body mass index (BMI), male sex, chronic obstructive pulmonary disease (COPD), advanced age, and family history [3]. According to Spector et al., 13 out of 1000 elderly patients have abdominal wall hernias [4], with inguinal hernias accounting for 75% of cases and bilateral hernias for 8%-30% [5].

The methodology for addressing bilateral inguinal hernias has undergone a complete transformation concerning the surgical technique employed and the considerations surrounding the repair procedure. Posterior inguinal wall reinforcement with Bassini or Shouldice was replaced with an anterior approach tension-free repair "Lichtenstein" or even laparoscopic repair, as the former techniques carry the risk of hernia recurrence because of the tension placed on the surrounding tissue. Sequential repair, which involves graded operations, with a 2-12-week interval, has been replaced by simultaneous repair for bilateral inguinal hernias [6,7]. Furthermore, simultaneous anterior mesh repair under local anesthesia and the Stoppa open preperitoneal approach using a large mesh implant are recommended for the simultaneous management of bilateral inguinal hernia repair [8-10].

The use of tacks (tackers) for mesh fixation has significantly reduced operative time and addressed the complications of traditional suture fixation, including persistent postoperative discomfort or neuralgia [11]. Elastic tacks also have better performance than metallic tacks in the short-term follow-up in terms of operative time, acute postoperative discomfort, and foreign body sensation. Additionally, the recurrence-free survival rate during the 40 months after hernia repair rose sharply from 71.5% to 82% when elastic tacks were used to secure the mesh rather than metallic ones [12].

## Case presentation

A 55-year-old man with a bilateral inguinal hernia for three years was referred by the general practitioner to the Federal Medico-Biological Agency Clinical Hospital (FMBA CH) No. 85, Moscow, Russian Federation, outpatient clinic. Primary arterial hypertension, chronic esophagitis, gastroesophageal reflux disease (GERD), duodenal ulcer, chronic prostatitis, and single-ventricular palpitations (SVP) were the comorbidities recorded during initial evaluation.

The general examination showed that the patient had a fair body build with no musculoskeletal abnormalities, no skin color changes, and no enlarged lymph nodes and was completely alert, conscious, and oriented. Vital signs were within normal range: blood pressure of 125/95 mmHg, body temperature of 37°C, respiratory rate of 18 beats per minute, and average-volume pulse of 75 beats per minute. Сhest examination showed that the respiratory sounds and respiratory movements were normal. The results of the cardiovascular examination showed normal peripheral pulsations, no congested neck veins, and normal heart auscultation without murmurs. Abdominal examination showed no ascites, no scars from prior surgeries, and a loose abdomen. The kidneys were pain-free and functioning normally. The patient has normal urinary function, with no signs of difficulty or discomfort during urination.

Local examination showed bilateral inguinal region hernial protrusions on both sides; the external inguinal ring was about 2.0 cm wide bilaterally, and the hernias were reducible, painless, and accompanied by an expansile impulse on cough. The hernial sac measured 5x4 cm.

A bilateral repair was decided upon by the Lichtenstein technique, using a polypropylene mesh for repair and elastic tacks (tackers) for mesh fixation. The patient was sent to the theatre with antithrombotic socks. Administration of intravenous fluids has been restricted to 700 mL in order to avoid post-operative urinary retention. A prophylactic preoperative antibiotic dose of 1 gm ceftriaxone was administered, and the type of anesthesia was general anesthesia.

**Figure 1:**
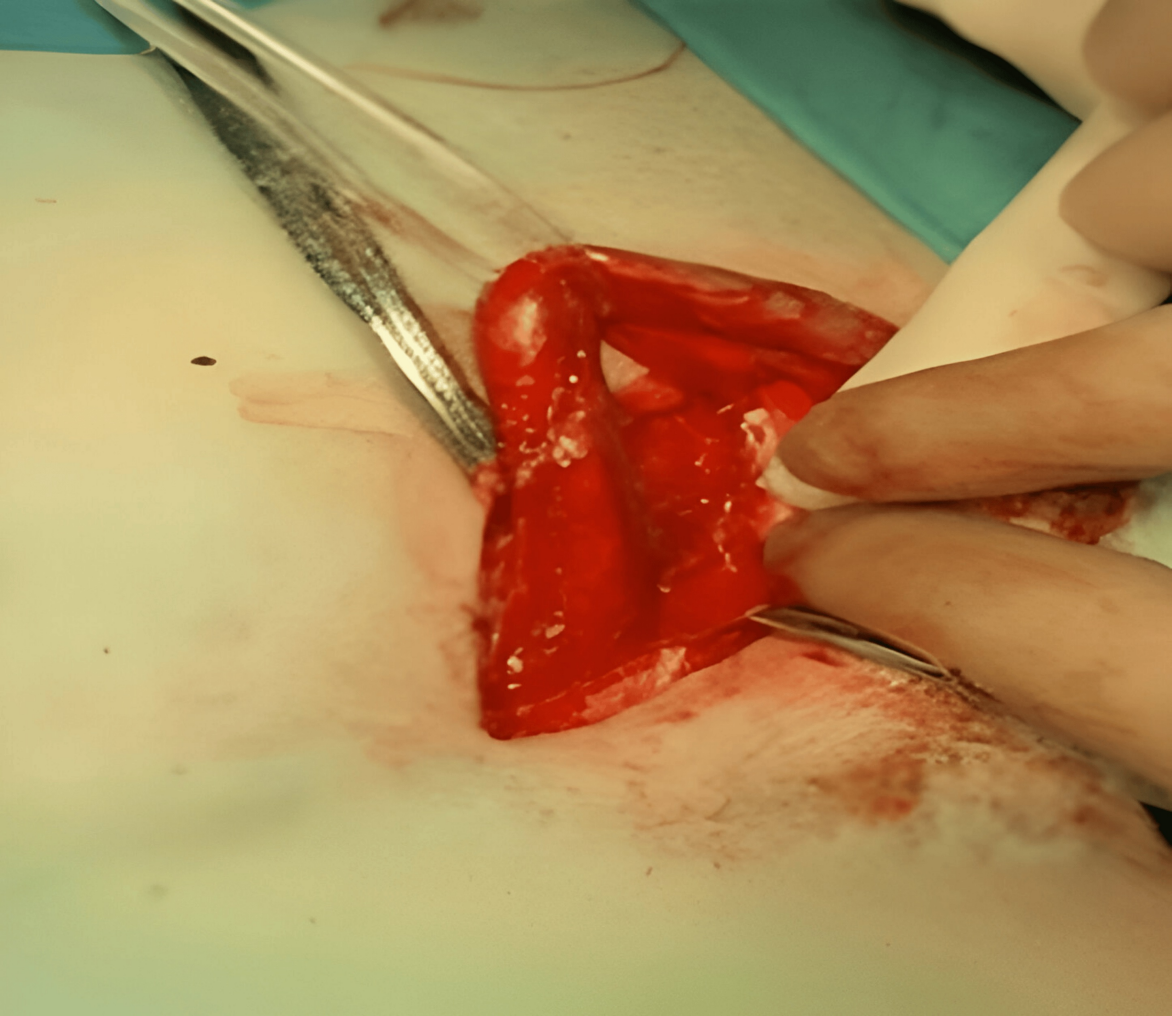
Encircling the spermatic cord using a Penrose drain exposing the hernial sac

A 4-cm inguinal incision was performed along the inguinal ligament, following Langer's line, after the inguinal area had been cleaned for two minutes. To minimize postoperative pain, edema, and ecchymosis, self-retaining retractors were used all over the wound to avoid excessive retraction of the wound margins. In order to avoid damage to the underlying ilioinguinal nerve, the left external oblique aponeurosis was carefully dissected along its fibers, starting at the external ring. The left external oblique aponeurosis and ilioinguinal nerve were then anesthetized. In order to preserve the genital branch of the genitofemoral nerve, we used a Penrose to encircle the spermatic cord and the hernia sac. We also placed our dissection plane near the pubic tubercle while encircling the cord (Figure 1).

**Figure 2:**
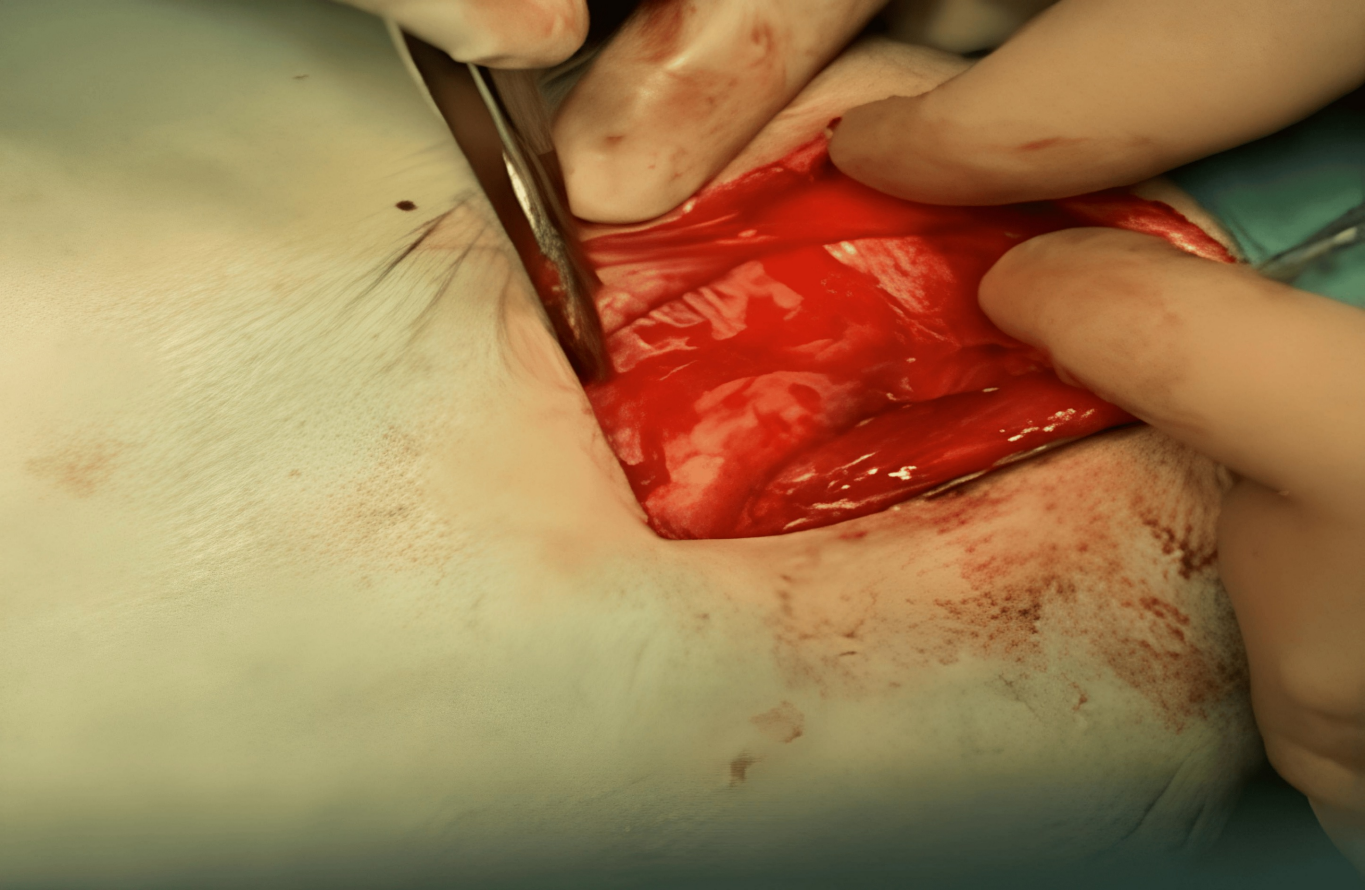
Hernial sac reduction (herniotomy)

**Figure 3:**
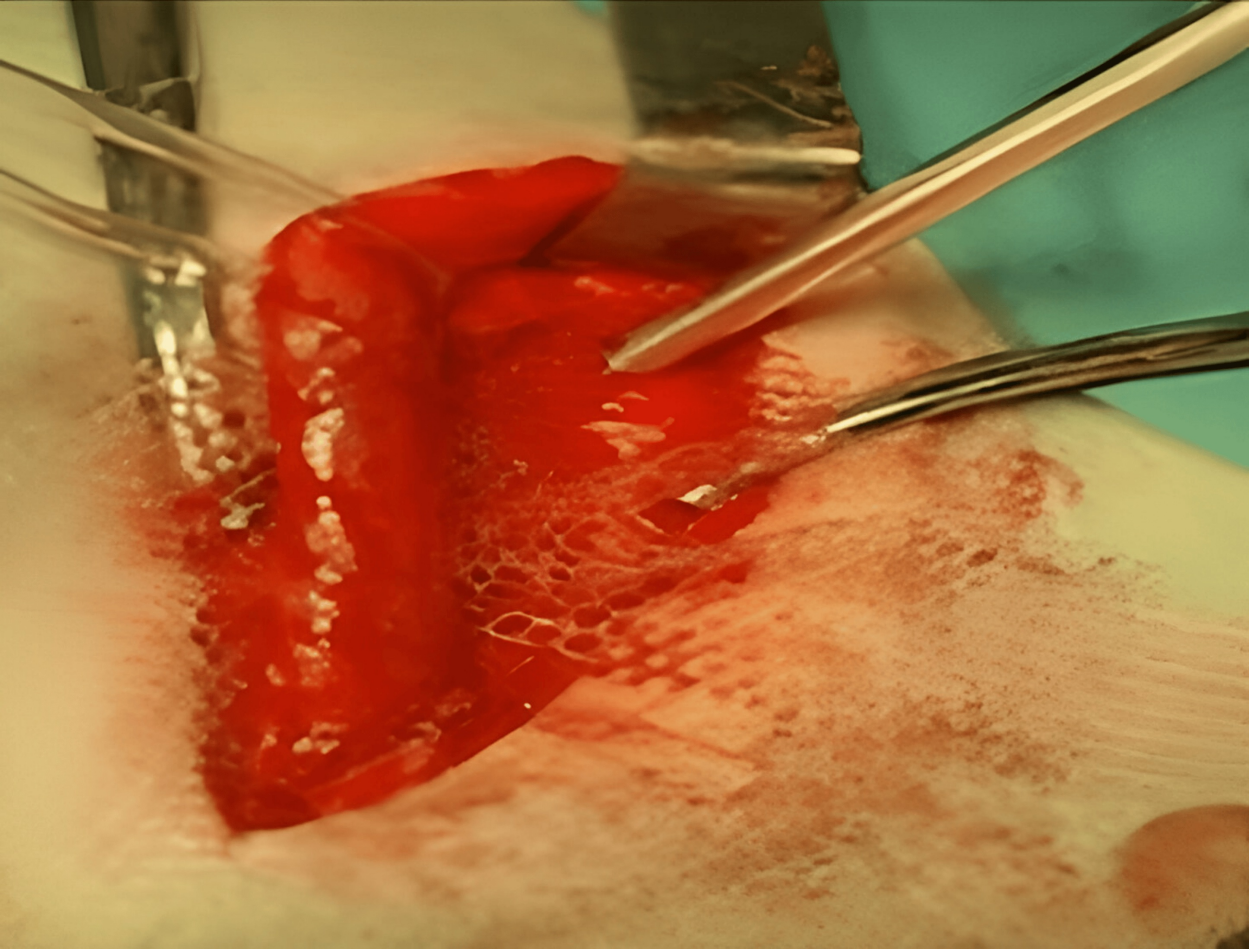
Application of the mesh implant

**Figure 4:**
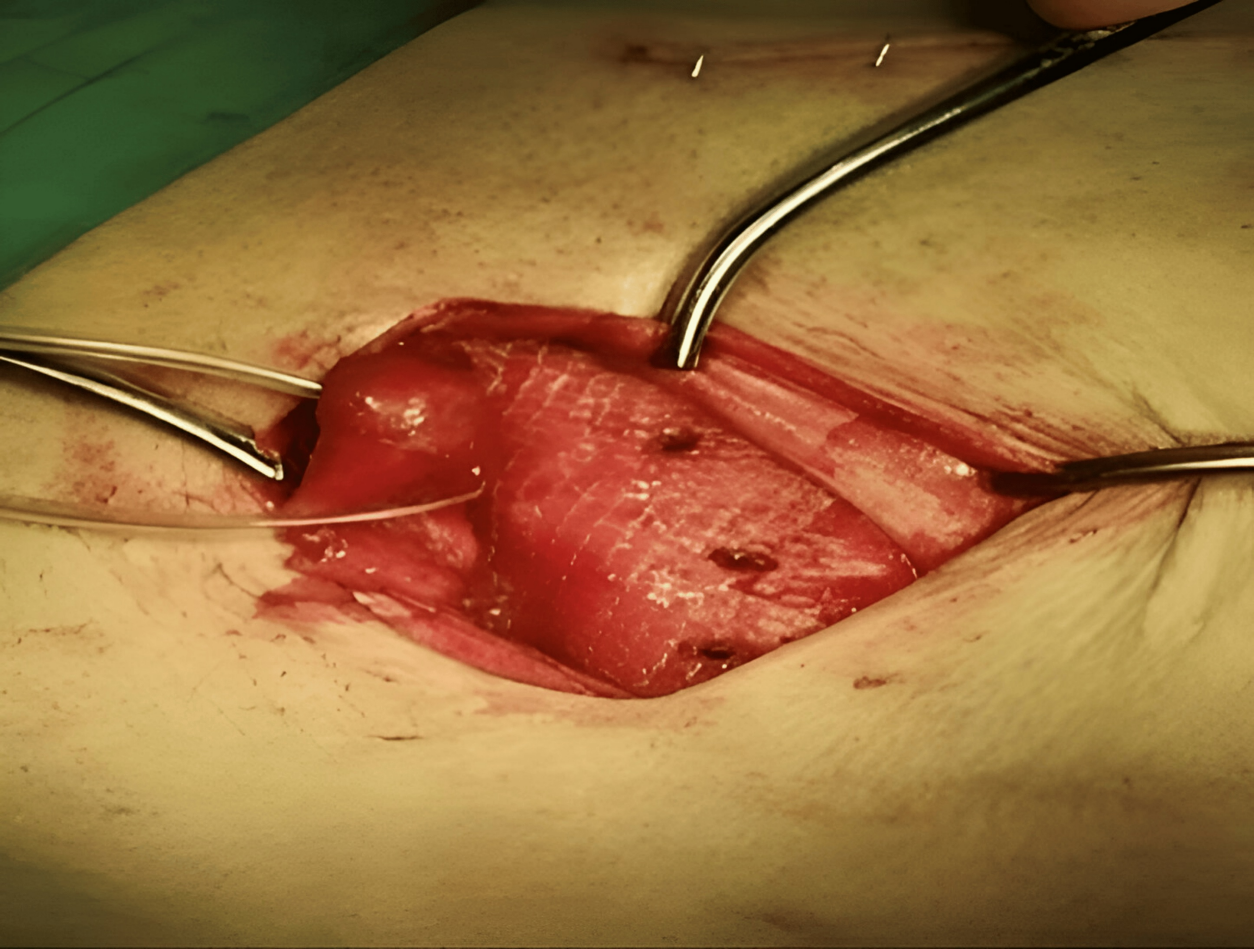
Layout of the mesh implant during fixation by herniostapler

Assuring the integrity of the genital branches of genitofemoral nerves, the ilioinguinal and iliohypogastric nerves were identified. Complete reduction of the peritoneum and all hernial contents back into the abdominal cavity was achieved through high dissection of the hernial sacs (Figure 2). Elastic tacks fixed by the COVIDIEN Absorbtack ^TM^ fixation device were used to secure a 6 x 11 cm polypropylene mesh to the inguinal ligament, starting from the pubic tubercle and moving towards the deep inguinal ring (Figures 3-4).

**Figure 5:**
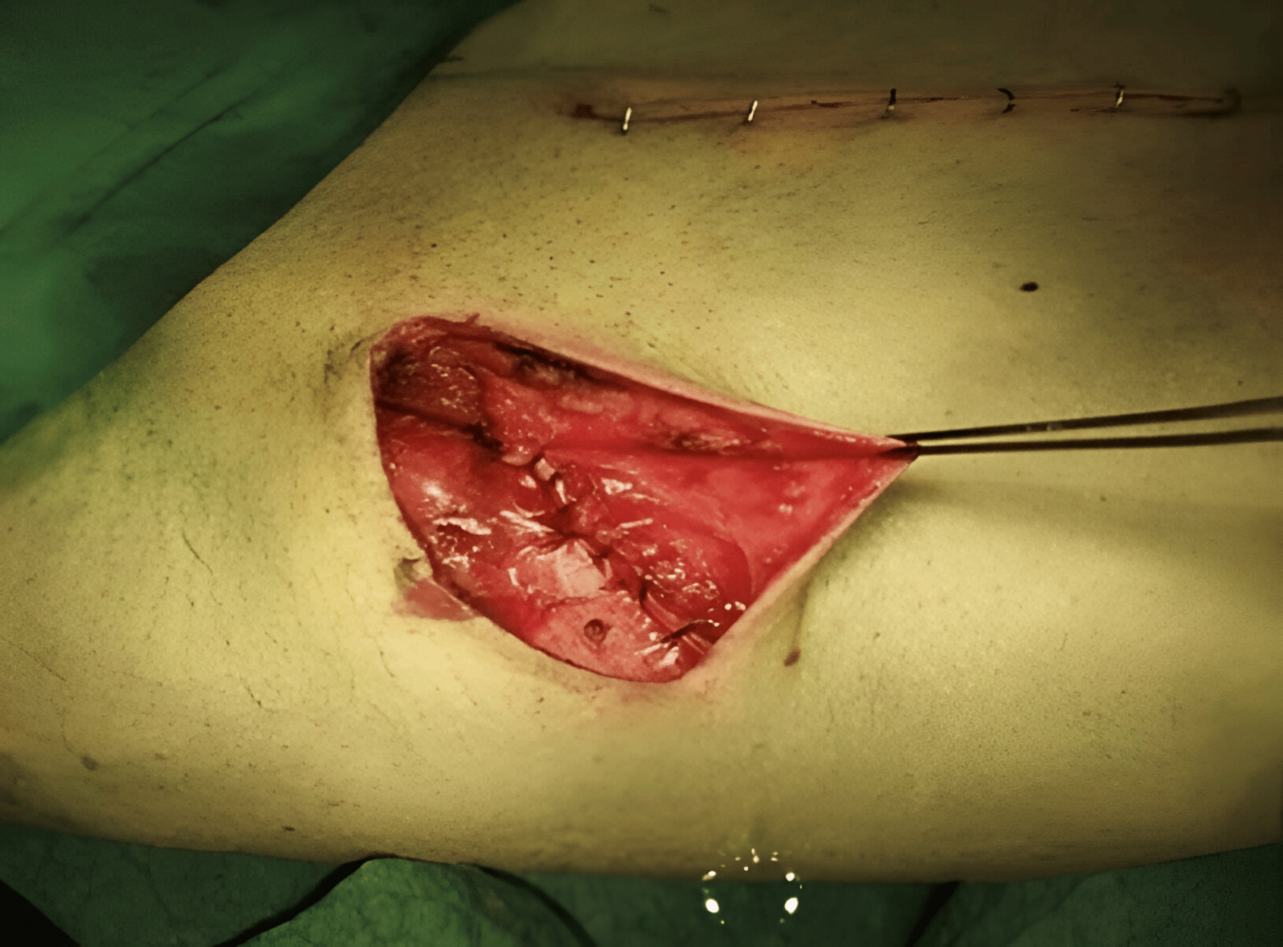
Suturing the muscles and aponeurosis

**Figure 6:**
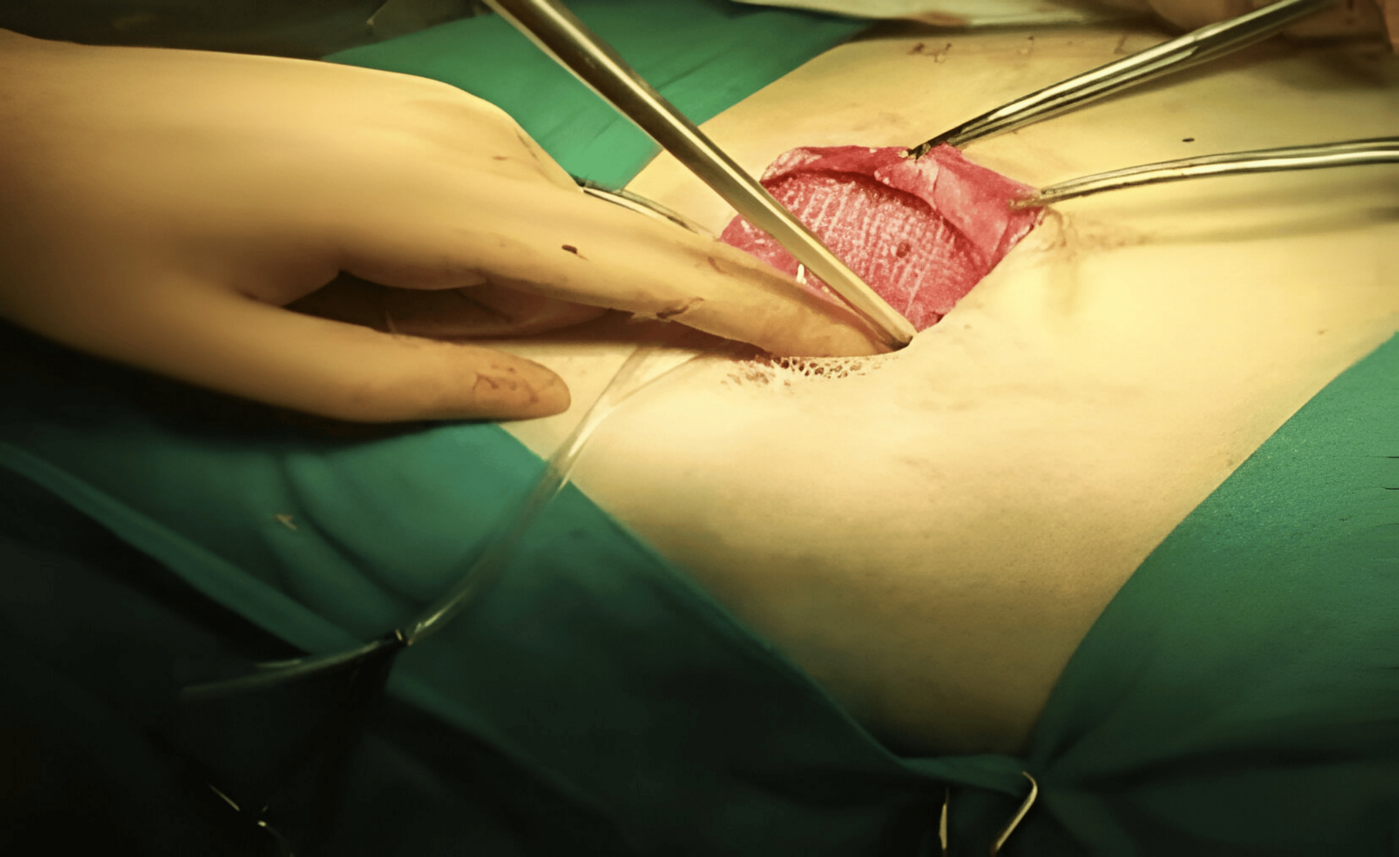
Fixing the mesh implant on the right side by tacks

The mesh was divided from lateral to medial; the lower tail is approximately one-third of the width of the upper tail, which is a reconstruction of the inner ring; and the spermatic cord is placed in this split. The external oblique aponeurosis has been reinforced with absorbable suture (Figure 5). The wound was closed layer by layer until the skin was approximated by metal staples, and then the same procedure was repeated on the other side (Figure 6).

A total of 40 minutes was the operative time. The results of the operation matched what was expected. Throughout the postoperative period, acute postoperative pain gradually subsided. Surgical dressing was applied once per day for a week. The patient had no chronic postoperative pain, seroma, foreign body sensation, or even recurrence during the short-term follow-up. Follow-up was scheduled every six months for the following five years. For the first 24 months, the follow-up results were as expected, with no complications, neuralgia, or recurrence.

## Discussion

Bilateral inguinal hernioplasty performed simultaneously improved surgical results. Comparing elastic tacks to metallic ones, mesh fixation with elastic tacks also reduces operative time, acute and chronic postoperative pain, and the incidence of foreign body sensation during the short-term follow-up [13].

Bilateral inguinal hernia management has been strongly controversial over the years. During the last three decades, staged operations with a 2-12-week interval were the most widely used approach because simultaneous repair was thought to increase the risk of complications and recurrences. Other options include simultaneous anterior mesh repair under local anesthesia or the open preperitoneal approach (Stoppa) with a large mesh. In addition to the potential for simultaneous repair of occult hernias, laparoscopic repair is another excellent option for minimally invasive treatment for bilateral hernias. Nevertheless, research supports the superiority of laparoscopic repair of bilateral inguinal hernias over open techniques, and Lichtenstein is not enough. Additionally, some research has shown that bilateral laparoscopic repair does not carry a higher morbidity rate than unilateral repair [7].

Therefore, in order to improve the results of the Lichtenstein technique using tack fixation, we conducted this clinical case report with the goal of obtaining the advantages of Lichtenstein-an easy-to-apply, cost-effective technique with a significantly low incidence of postoperative complications such as recurrence-so that it is considered the gold standard choice for open inguinal hernia repair. Additionally, we aimed to achieve the benefit of using herniostaplers by tacks (tackers), which saves operating time and lowers the incidence of postoperative pain resulting from classical suture fixation [14,15].

The analysis of bilateral open inguinal hernia repair and Rives-Stoppa repair techniques yielded important information on treating bilateral inguinal hernias. Both approaches have particular advantages and disadvantages that impact operative time, postoperative pain, and recurrence rates. While bilateral open inguinal hernia repair is more straightforward and comfortable, the Rives-Stoppa operation may offer advantages in terms of reduced recurrence rates and postoperative complications. These findings have significant implications for clinical practice, highlighting the importance of selecting the optimal surgical approach by considering patient-specific factors, surgeon experience, and hospital resources. More research is required to conduct long-term comparative studies that evaluate postoperative outcomes other than recurrence rates and look into cutting-edge surgical techniques and materials. Such studies have the potential to improve patient outcomes, reduce healthcare costs, and improve inguinal hernia repair [16].

Due to the disagreements among many authors, our team chose to proceed with this case using a modified open inguinal hernioplasty technique in an effort to improve outcomes.

## Conclusions

We observed that the operative time, postoperative complications, and hospital stay were significantly reduced when we performed Lichtenstein hernioplasty for bilateral inguinal hernioplasty at the same time. This will allow us to continue this trial as a research topic in order to obtain clinical recommendations for the surgeons in our area of interest. For patients with bilateral inguinal hernias, it would also be very meaningful to compare the use of traditional open inguinal hernioplasty with modified alternatives. Additional questions based on the surgeon's experience and the hospital's capacity could be useful in a comparative analysis of open versus laparoscopic techniques. We can recommend tack fixation as a modification for open inguinal hernia repair as it reduces operative time and may reduce postoperative pain based on mesh fixation techniques for patients with bilateral inguinal hernias in the same set, based on previously discussed points.
